# Development of a Statistical Workflow for Screening Protein Extracts Based on Their Nutritional Composition and Digestibility: Application to Elderly

**DOI:** 10.3390/foods9101499

**Published:** 2020-10-20

**Authors:** Angéline Duval, Thierry Sayd, Laurent Aubry, Claude De Oliviera Ferreira, Vincenza Ferraro, Véronique Sante-Lhoutellier

**Affiliations:** QuaPA, Quality of Animal Products, INRAE, 63122 Saint-Genès-Champanelle, France; angeline.duval@inrae.fr (A.D.); thierry.sayd@inrae.fr (T.S.); laurent.aubry@inrae.fr (L.A.); claude.ferreira@inrae.fr (C.D.O.F.); vincenza.ferraro@inrae.fr (V.F.)

**Keywords:** statistical analysis workflow, in vitro digestion, elderly, protein extracts

## Abstract

The objective of the study is to develop a workflow to screen protein extracts and identify their nutritional potential as high quality nutritional culinary aids for recipes for the elderly. Twenty-seven protein extracts of animal, vegetable, and dairy origin were characterized. We studied their fate by monitoring static in vitro digestion, mimicking the physiological digestion conditions of the elderly. At the end of the gastric and intestinal phase, global measurements of digestibility and antioxidant bioactivities were performed. The statistical analysis workflow developed allowed: (i) synthesizing the compositional and nutritional information of each protein extract by creating latent variables, and (ii) comparing them. The links between variables and similarities between protein extracts were visualized using a heat map. A hierarchical cluster analysis allowed reducing the 48 quantitative variables into 15 qualitative latent variables (clusters). The application of the k-means method on each cluster enable to classify the protein extracts by level. This defined level was used as categorical value. Multiple correspondence analysis revealed groups of protein extracts with varied patterns. This workflow allowed the comparison/hierarchization between protein extracts and the creation of a tool to select the most interesting ones on the basis of their nutritional quality.

## 1. Introduction

The aging population will become a major societal issue as the number of people over 60 years of age continues to grow. At the global level, estimates put the population over 60 years of age at 2 billion people in 2050 compared to 629 million today. At the same time, the risk of undernutrition and deficiency for the elderly affects between 4% and 10% of people living at home, between 15% and 38% of people living in nursing homes and up to 70% in hospitals [1]. There is therefore a need to supplement the diet of the elderly. It is accepted that protein requirements for them are about 20% higher to maintain body protein mass [1] and specific amino acids such as leucine play a beneficial and protective role in terms of muscle mass and function [2]. In addition, a diet rich in protein and specific amino acids is important for maintaining muscle mass in the elderly, who are often subject to low-grade inflammation [3]. It was shown that antioxidant supplementation improved the anabolic response to leucine of old muscle and reduced inflammation [4]. Older people regularly suffer from sarcopenia. Sarcopenia is defined as a progressive and generalized decline in muscle mass, strength, and physical performance in geriatric patients. In addition, aging is accompanied by changes, including alterations and the deterioration of intestinal functions, such as less secretion of digestive fluids and enzymes, and a higher pH in the stomach [3,4,5,6]. As a result, food is less well assimilated by the body, so it is important that the proteins and amino acids provided through supplementation are highly bioavailable. To prevent sarcopenia, the most effective approach is a combination of physical exercise and an adequate intake of amino acids, and macro and micro nutrients [7,8,9].

The digestibility of a protein depends on its origin and more precisely on its amino acid composition. The PDCAAS (which is protein digestibility corrected amino acid score) has been the reference in the notion of protein quality and recommended by the FAO and WHO for more than 20 years. The PDCAAS considers the essential amino acid profile of proteins—and thus makes it possible to identify the limiting factor—and the overall digestibility of the protein. The FAO has recommended replacing the PDCAAS with the digestible indispensable amino acid score (DIAAS). The new recommended method is more precise, as it considers the digestibility of each essential amino acid [10]. Other methods approaching digestibility such as OPA (ortho-phthaldialdehyde) spectrophotometric assay or fluorescamin fluorimetric assay [11] evaluate the degree of protein hydrolysis through the amount of NH_2_ groups released.

The quality of a protein depends on its composition in essential amino acids but also on the anti-nutritional factors that may be present and thus limit protein digestion. With this new score of DIAAS, milk proteins reached high values (>100%), which is twice as high as that of peas (~64%) and three times as high as that of wheat (~40%) [12,13]. Proteins of vegetable origin are often incomplete in amino acids, for example cereals are deficient in lysine whereas legumes are deficient in sulphur amino acid. In addition, some antinutritional factors (ANFs)—such as phytate, phenolic compounds, tannins, and fibers—are present in plant foods and known to contribute to the low bioavailability of some minerals essential for elderly, like iron and zinc. Various processing technologies (mechanical treatments, soaking, germination, fermentation, and heating) can be applied to improve the mineral bioaccessibility [14]. For example, heat treatments reduce the content of ANFs up to 40%, as reported for legumes [15].

With plant proteins, it is thus necessary to combine them to balance essential amino acids. The PNNS (Programme National Nutrition Santé, French nutrition program) [16] recommends the diversification of protein sources in order to meet the complementary nutritional requirements for overall balance (vitamins, minerals, etc.). This also leads to the improved use of natural resources, especially plant proteins. Continuing along these lines, our objective was to select protein ingredients (i.e., protein extracts) that would meet the nutritional requirements of the elderly, and be well digested in order to be effectively assimilated by the body.

In addition, bioactive peptides are generated from different food sources during gastrointestinal digestion. Bioactive peptides have a variety of targets such as immune, cardiovascular, digestive and endocrine systems in the human body [17,18,19,20]. In the case of personalized nutrition for elderly, such properties may be considered in the nutritional value of protein sources.

To this end, multivariate analysis is often used to integrate different types of data and extract meaningful information. Multivariate studies are increasingly being developed, particularly to study omics datasets, but are still rare, especially in the field of nutrition [21,22].

Therefore, the aim of this study is to develop a statistical analysis workflow from compositional and nutritional properties as an efficient tool to compare all the protein extracts, all things being equal.

## 2. Materials and Methods

### 2.1. Protein Ingredient

The 27 protein ingredients (Table 1) of animal (*n* = 9), plant (*n* = 9) and dairy (*n* = 9) origin were provided by Solina (Bréal-sous-Montfort, France), Sodiaal (Paris, France), Diana Food (Val-Couesnon, France), Cooperl (Lamballe, France), and Adria (Quimper, France).

### 2.2. Proximate Analysis

The dry matter (DM) content was determined after heating the protein extracts at 100 °C for 16 h in an oven. The protein content was determined by the method of Dumas [23] using a conversion factor of 6.25. In accordance with ISO 16634, this factor is used for food products of animal origin, pulses, and oilseeds. The results are presented in g of protein per 100 g of ingredient. Amino acid analysis was performed with an amino acid analyzer (L-8900, Hitachi, Paris, France), using the same methods as described in Margier et al. [24]. The results are expressed as the percentage of amino acid of dry matter.

Carbohydrates were determined using the Du Bois method [25], based on UV/VIS spectrometry. The results are expressed in g of carbohydrates/100 g of ingredients.

Essential macro-minerals (K, Ca, Mg, and Na) and trace elements (Cu, Cr, Fe, and Zn) with a proven risk of deficiency in the elderly were quantified by inductively coupled plasma spectrometry (ICP-AES) following the Poitevin method [26]. All the results for the macro and micro elements are expressed in ppm.

Vitamin C in the protein extracts was determined by a fluorimetric method [27]. The method is based on the condensation reaction between ascorbic acid and o-phenylenediamine (OPDA) in the absence of an oxidant. Vitamin A was assessed differently, depending on the origin of the protein extracts. Indeed, it is found in the form of retinol in animal tissues, while in plant tissues it is found in the form of provitamin A (precursors of vitamin A). Retinol was determined as retinyl acetate (retinol ester) by the Bayfield method [28]. Provitamin A was determined as beta-carotene using the method of Biswas et al. [29]. Vitamin C is expressed in mg/g of protein extract while vitamin A is expressed in µg/g of protein extract.

In addition to the composition of the protein extracts, the level of oxidation of lipids and proteins was also evaluated as it can influence the bioavailability of these macronutrients. The oxidation of proteins is determined by measuring total carbonyls with the DNPH method [30] and expressed in nM hydrazones/mg protein. In order to determine the oxidation of lipids, the method of Lynch and Frei [31] was used. This method is based on the quantification of secondary compounds (TBARS). The results are expressed in mg of malondialdehyde per kg of protein extract. Each measurement was assessed in triplicate.

### 2.3. In Vitro Digestion of Protein Extracts

The protein ingredients were subjected to gastric and intestinal conditions according to the standardized static digestion method developed by Minekus et al. [32], with slight modification in order to mimic elderly physiological conditions, i.e., gastric pH and enzyme quantity [33]. The buffer used in the gastric phase is composed of 6.9 mmol/L KCl, 0.9 mmol/L KH_2_PO_4_, 25 mmol/L NaHCO_3_, 47.2 mmol/L NaCl, 0.1 mmol/L MgCl_2_(H_2_O)_6_, 0.05 mmol/L (NH_4_)_2_CO_3_, and 0.15 mmol/L CaCl_2_ 2H_2_O with a pH adjusted at 2 with HCl. The buffer used in the intestinal phase is composed of 6.8 mmol/L KCl, 0.8 mmol/L KH_2_PO_4_, 85 mmol/L NaHCO_3_, 38.4 mmol/L NaCl, and 0.33 mmol/L MgCl_2_(H_2_O)_6_, with a pH adjusted at 7 with NaOH. The pH of the empty stomach of the elderly is 3, unlike in younger adults, in which it is 2. The gastric phase lasted 1 h, while the intestinal phase took 2 h. The quantities of enzymes were also reduced compared to Minekus et al. protocol—in the gastric phase: pepsin (1200 U/mL); and in the intestinal phase: trypsin (67 U/mL), chymotrypsin (16.75 U/mL), and lipase (400 U/mL). For each protein extracts digestion, the amount of material dispersed corresponded to 5 g of protein. For each protein extract, three digestions were carried out. Samples were taken at the end of the gastric phase and the intestinal phase, and TCA (final vol. 15%) was added. The samples were then centrifuged and the supernatant was stored at −80 °C until analysis. Bioaccessibility was assessed using several methods. Firstly, by the Biuret method, which allows the determination of proteins on the basis of peptide bonds [34]. Two methods were used to assess the degree of protein hydrolysis obtained: the Direct Detect^®^ spectrometer (Merck, Billerica, MA, USA) and fluorescamine. Direct Detect^®^ allows determining the total peptides [35] by infrared while the fluorescamine assay measures the N terminal function of amino acids and peptides [11]. Each measurement was performed in triplicate.

### 2.4. Bioactivity

Four different methods were used to evaluate the antioxidant potential of peptides from the supernatant obtained as described above. Trolox^®^ ((6-hydroxy-2,5,7,8-tetramethychroman-2-carboxylic acid; Aldrich Chemical Co., Gillingham, Dorset, UK) was the reference solution for each of the assays. The first method was a 2,2′-azinobis(3-ethylbenzothiazoline-6-sulphonic acid, Roche Diagnostic GmbH, Mannheim, Germany)) free radical scavenging activity test (ABTS) according to the method of Re et al. [36], with slight modification of the volume of sample pipetting (100 µL instead of 10 µL). The result obtained was the percentage of inhibition of the ABTS radical. Antioxidant activity on lipophilic radicals was measured by the method of Brand-Williams et al. [37], using the stable radical DPPH (2,2-diphenyl-1-picrylhydrazyl). The results are expressed in percentage of inhibition of DPPH radical. The antioxidant potential of protein extracts has also been estimated based on their ability to chelate iron (FRAP) [38]. This method assesses the ability to reduce the Fe(III)-2,4,6-Tri(2-pyridyl)-s-triazine (TPTZ) complex to Fe(II)-TPTZ. Therefore, the results represent the antioxidant ability to reduce ferric ions (% inhibition). Finally, the determination of oxygen radical absorption capacity (ORAC) was carried out according to the method described by Ou et al. [39] and adapted by Da’valos et al. [40]. The result are expressed in µmol Trolox equivalent/mg peptide digestate.

### 2.5. Statistical Analysis

The statistical analysis was carried out with STATISTICA software (version 13.3) from TIBCO Software Inc. (Palo Alto, CA, USA), Permut matrix (software version 1.9.3.0, [41]) and R software (version 3.6.3). The values for each variable were reported as the mean ± standard error of the mean (SEM) of three independent repetitions. Experimental data were subjected to heat map clustering (Permut matrix software)) using the Pearson distance and Ward’s inertia. Then, a hierarchical cluster analysis (HCA) and a classification by k-means were performed on the data (STATISTICA software). Finally, a multiple correspondence analysis (MCA) was performed on the Factoshiny package of R software.

## 3. Results

The results of the composition of each protein extract are summarized in Table 2, which therefore provides the composition of macro and micronutrients as well as the oxidation of lipids and proteins of each protein extract. Plant extracts contain the most carbohydrates, and complex sample A5 (Pork liver) in particular has a very high protein and lipid oxidation rate. This powder also has a high iron content.

Table 3 shows the amino acids contained in the samples. In general, protein extracts from animal origin were those that contained the highest protein content. Protein extracts P1 (fava bean) and P2 (fermented fava bean) contained less protein and amino acids compared to the other extracts. Protein extracts D4 (whey protein), D5 (whey protein), D9 (whey protein), and P4 (pea) contained the highest leucine content, which is very important for maintaining muscle mass. It can be seen that samples A4 (pork collagen), D1 (cheese powder), D3 (60% micellar casein concentrate), P1 (fava bean), and P2 (fermented fava bean) were deficient in leucine and branched AA. Finally, the protein extracts that were the most deficient in aromatic amino acids were D2 (80% serum protein concentrate), P1 (fava bean) and P2 (fermented fava bean) whereas for sulfur amino acid it is the protein extracts A4 (pork collagen) and A7 (chicken broth) that were deficient.

### 3.1. Bioaccessibility and Bioactivity

According to Table 4 and Table 5, globally protein extracts of animal origin are more hydrolyzed in the gastric phase. However, protein extracts obtained from chicken or pork broth (A7 and A3, respectively) exhibited a low value of bioaccessibility measured through NH_2_ group release, even under the value of plant or dairy powder. In the gastric compartment, protein hydrolysis is due to pepsin action. The cutting site of pepsin is after an aromatic amino acid. Therefore, in an attempt to possibly explain the difference due to protein source, the content of all aromatic amino acids was compared regarding the origin (plant, dairy, or animal). No significant difference was observed (data not shown). In a global manner, protein extracts of plant origin were less hydrolysed in the gastric and intestinal phase. D1 (cheese powder) and P1 (fava bean) were essentially found as less hydrolysed samples in the intestinal phase. Fermentation of fava bean (P2) did not modify this observation. Antioxidant bioactivity was recorded in the gastric phase. The animal samples obtained from pork liver (A5 and A6) exhibited the highest antioxidant activity, in the same range than fava beans (P1 and P2) and sunflower seeds (P9). Chicken and pork broth (A7 and A3, respectively) displayed a low antioxidant activity. Both plant and animal samples presented heterogenous antioxidant bioactivity, may be explained by the presence of phenolic compounds, able to chelate metals. On the contrary, the antioxidant activity of the dairy protein extract in the gastric phase was rather similar between samples, in average 40% less of the mean of plant or animal samples. Similar results were observed in the intestinal phase.

Many parameters are involved in the digestion process of protein extract. This marks the limit of reasoning variables per variables, with a large set of samples. Therefore, multivariate analysis is required to integrate different types of data and to deal with the data complexity to extract meaningful information.

### 3.2. Global Visualization of Variables and Protein Extracts

**Figure 1:**
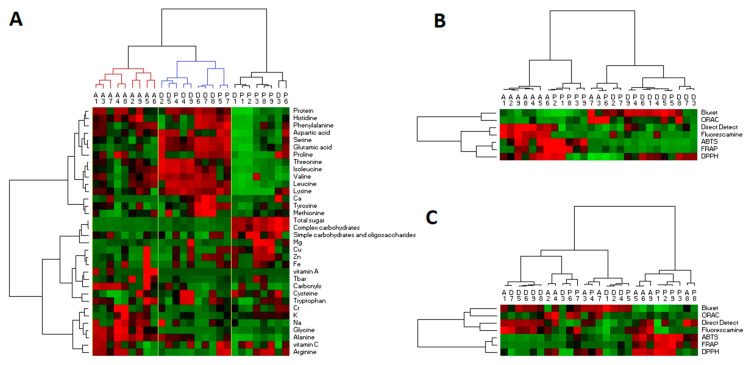
Heat map clustering on the composition of the protein extracts (**A**), in the gastric (**B**), and intestinal phases (**C**). The data were standardized. Double classification using Ward’s method and Pearson distance.

Heat map clustering allows grouping protein extracts according to their compositional characteristics (e.g., some AA, carbohydrates, etc.). For example, in Figure 1A the protein extracts D1 (cheese powder), P1 (fava bean), and P2 (fermented fava bean) contain large amounts of total sugar, simple and complex carbohydrates, and Mg. These variables represent a carbohydrate cluster.

Figure 1A also shows that animal protein extracts are linked together, which means that in terms of composition they are similar extracts.

Figure 1B,C shows that animal samples appear to be better digested and develop more antioxidant activity in the gastric and intestinal phases. In addition, ABTS, FRAP, and DPPH are related, as are the results obtained by the Direct Detect^®^ method and fluorescamine. The interpretation of the data by heat map clustering is difficult because there are many variables, and our aim is to gather composition and digestion data. Thus, the number of variables must be reduced.

### 3.3. Reduction: Association of Quantitative Variables by Clustering

To reduce the number of variables we start by analyzing the variables using a hierarchical cluster analysis (HCA). In order to gather the variables that are related to each other in a cluster. This new group of variables is defined by a new name and represents a latent variable.

**Figure 2:**
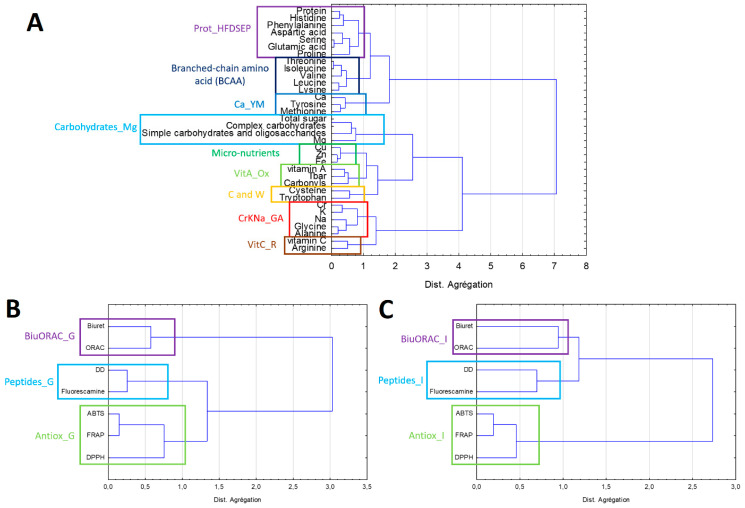
Hierarchical cluster analysis (HCA) of the composition of the protein extracts (**A**) of the gastric (**B**) and intestinal phases (**C**). Boxed variable groups represent the newly created latent variables. The data were standardized. Double classification using Ward’s method and Pearson distance.

Figure 2A shows the classification of the composition data. This allows us to visualize which variables are related and thus bring them together to create new variables called latent variables. Nine latent variables were created from the 34 composition variables. In Figure 2B,C, Direct Detect^®^ results are related to the results obtained by the fluorescamine method. Both methods measure peptide levels. The ABTS, DPPH, and FRAP methods measure the antioxidant activity of bioactive peptides and are linked together. As in the intestinal phase, three latent variables were created from seven variables in the gastric phase. To sum up, the 48 initial variables were merged into 15 latent variables.

### 3.4. Classification of Protein Extracts to Obtain Categorical Value to Each Latent Variable

The second step of the analysis consists of attributing for each protein extracts a categorical value for each cluster (latent variable) defined above. The k-means method was applied to the quantitative variables of a cluster, allowing classifying the protein extracts into classes. Then, a categorical value (high, medium, and low) was assigned to each protein extract.

**Figure 3:**
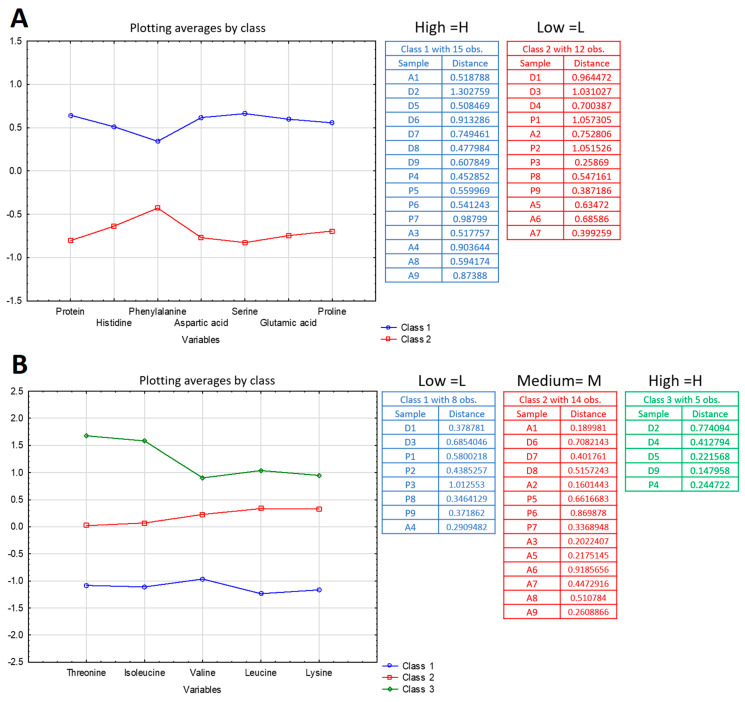
Kmeans performed on the latent variable Prot_HFDSEP (**A**) and TIVLK (**B**). The latent variable Prot_HFDSEP contains two levels (high and low). The latent variable TIVLK contains three levels (high, medium, and low).

Figure 3A corresponds to the results obtained for the latent variable Prot_HFDSEP, which consists of the content variables of histidine, phenylalanine, aspartic acid, serine, glutamic acid, and proline. The protein ingredients can be divided into two classes using the k-means method. The first class includes ingredients with a high level and the second class includes protein extracts with a low level in its components.

Figure 3B corresponds to the results obtained for the latent variable TIVLK which is composed of the content variables of threonine, isoleucine, valine, leucine, and lysine. The protein extracts are divided into three classes: high, average, and low level in these amino acids.

This method is used for all the latent variables created with HCA. All the data obtained are gathered in Table 6.

### 3.5. Synthesis

The results obtained after the creation of latent variables by HCA and then class variables by the k-means method are summarized in Table 6. From the 48 initial variables, 15 latent variables were created, with two or three levels (high, medium, or low).

In order to highlight associations between protein extracts and link with latent variable, a multivariate correspondence analysis (MCA) was conducted (Table 6). If a class was only composed of two protein extracts the latent variable was not used for the MCA because it would be too discriminating. Thus, the latent variables vitA_Ox and CrKNa_GA were not considered.

**Figure 4:**
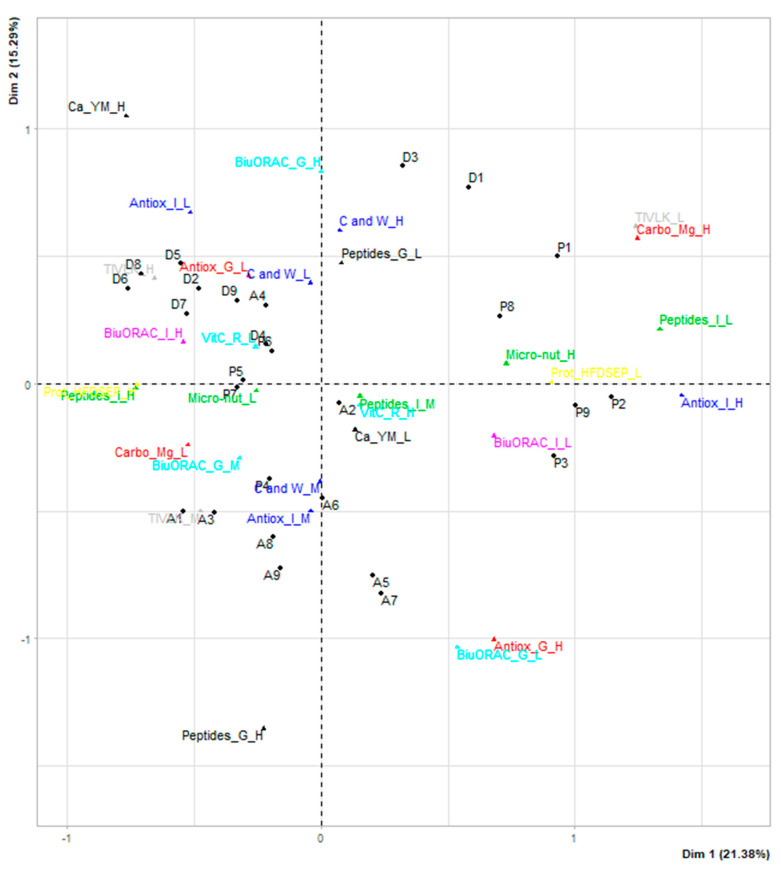
Correspondence graph of individual variants of latent variables. Individuals: protein extract. 13 latent variables and 30 variants.

The resulting graph is shown in Figure 4. The percentage variance expressed is 21.38% for the first axis and 15.29% for the second axis. Both factors explained 36.67% of the total system inertia. Dimension 1 opposed individuals such as P3 (hemp), P2 (fermented fava bean), and P9 (sunflower) to individuals such as D8 (milk protein concentrate), D6 (calcium caseinate), and D5 (whey protein). The group in which individuals P3 (hemp), P2 (fermented fava bean), and P9 (sunflower) are found have the following characteristics: a high level of carbohydrates, a low level of TIVLK, a low level of protein and antioxidant activity (ORAC) in the intestine, a high level of antioxidant activity in the stomach and intestine. The individuals D8 (milk protein concentrate), D6 (calcium caseinate), and D5 (whey protein) shared a high level of intestinal peptide, a high level of protein and HFDSEP and a low level of antioxidant activity in the intestine. On the other hand, dimension 2 opposed individuals such as and D8 (milk protein concentrate), D6 (calcium caseinate), D1 (cheese powder), D3 (60% micellar casein concentrate), and D5 (whey protein) to individuals such as A7 (chicken broth) and A5 (pork liver). D8 (milk protein concentrate), D6 (calcium caseinate), and D5 (whey protein) are characterised as above. D1 (cheese powder) and D3 (60% micellar casein concentrate) are characterized by their high level of cysteine and tryptophan, whereas, A7 (chicken broth) and A5 (pork liver) share a high level of gastric peptides. However, some protein extracts remained undifferentiated, located near the centre of the graph, such as P4 (pea), P6 (soybean), and A2 (pork liver).

## 4. Discussion

All the protein extracts analysed contained several vitamins and organic and mineral nutrients; however, some contained more of certain minerals than others, or were less digestible, etc. We observed that the composition of the protein extracts was linked to their origin. For dairy products, calcium caseinate (D6) and casein rennet (D7) showed the highest level of calcium, as expected. Calcium is an important mineral for the development strong bones by proper intake in young people, and for keeping the bones of the elderly strong and healthy, by preventing a variety of bone-related illnesses, such as osteoporosis. The review of Philips et al. [42] highlighted that whey proteins were the best for supporting muscle protein synthesis due to their high leucine content compared to milk and soy proteins. The absence or insufficient quantity of an essential amino acid is enough to disrupt protein synthesis. It is therefore the balance between the different essential amino acids (histidine, isoleucine, leucine, lysine, methionine, phenylalanine, threonine, tryptophan, and valine) that will be the first factor of dietary protein quality. Recently Reynaud et al. [13] evaluated the digestible indispensable amino acid scores (DIAAS) of pea emulsion (40–64%), tofu and soya milk (78–116%). Interestingly those results concerning soya products were comparable to those reported for milk (≥100%) [12] and meat protein (80–99%) [43]. However, no information about a possible bioactivity was reported.

Diets rich in plant foods are increasingly recommended to reduce the risk of cardiometabolic diseases because of strong evidence that fruit, vegetables, legumes, and seeds are protective. In this study, plant-based protein extracts presented slower hydrolysis in the upperpart of our in vitro digestive system than animal and dairy products, which cannot be explained by less aromatic amino acid content. Antinutritional factors could be involved such as phytic acid or protease inhibitors, but they were not analysed in this study. The literature showed that mechanical treatment, heating, and fermentation of plant-based products, to cite a few, could partly overcome this adverse effect [15]. Moreover, Bax et al. [44] reported that the speed of pepsin digestion in the gastric compartment was explained by an enhanced enzyme accessibility due to protein denaturation. According to Dangin et al. [8] and Serafini et al. [9], proteins have specific absorption rates based on amino acid composition, which characterizes the anabolic properties of ‘fast’ or ’slow’ protein absorption. The rapidity of absorption of dietary amino acids by the intestine is crucial, because it influences the rate of postprandial protein synthesis, and therefore muscle mass accretion. Moreover, the composition of plant-based protein extracts contained lower amounts of lysine, methionine and/or leucine. Besides the rate of digestion, plant-based protein extracts generally contained more complex sugars. Fibers are important in the diet because they contribute to the proper functioning of the gut, promoting transit and so forth, but some fibers can act as an anti-nutritional factor, i.e., reducing the digestibility of proteins.

In addition to their high nutritional value, proteins can be precursors of bioactive peptides released during digestion, acting locally or on other organs. These peptides are characterized by their beneficial properties on key body functions. Indeed, natural antioxidants are beginning to be considered for the treatment of cellular degeneration because they inhibit or delay the oxidation process by blocking both the initiation and propagation of oxidative chain reactions [45]. All the protein extracts analyzed exhibited antioxidant activity with various degree. In the plant samples, fava bean and sunflower protein extracts (P1, P2, and P9, respectively) displayed high antioxidant bioactivity especially during the intestinal phase. One explanation could be the presence phenolic compounds, such as chlorogenic acid, predominant in sunflower kernel [46].

Many parameters are involved in the digestion process of protein extract to determine its nutritional value. This leads to studying a few protein-based food or a limited number of variables in a same row in the literature. In the future, the promotion in the diet of plant-based protein foods will increase as a transition towards more sustainable food consumption, particularly a substitution of animal protein with plant-based protein sources [47]. The in vitro approach developed in this study allows to screen samples for elderly before any in vivo determination of the true ileal digestibility of amino acids, for example, which requires significant resources [13].

In order to compare the different protein extracts using all the variables, it was necessary to develop a statistical workflow. Secondly such an approach can also help to better combine different protein sources to improve the essential amino acid profile, bioactivity, digestibility, and fiber content.

The capacity to compile large amounts of different types of data has become possible thanks to the development of omics data sets. Multivariate analysis is often used to integrate different kinds of data and attempt to extract meaningful information. Graphical representation is also part of the challenge to aid interpretation [48]. Basically, we started with a classical statistical analysis of data visualization (heat map) in two dimensions, and variable grouping was performed in order to reduce data. The HCA method builds the hierarchy from the individual elements (i.e., variables) by progressively merging clusters. In our study, three sets of variables were used and three HCAs were built: the first set on composition, the second on digestive variables in the gastric compartment, and the third on the same variables but in the intestinal compartment. Then, the next step was to determine which elements should be merged in a cluster. For the first set the 34 variables were merged into 9 clusters. For the second and third sets, the seven variables were merged into three clusters. Each cluster gave rise to a latent variable, also called categorical variable. The application of the k-means algorithm was then used to group the input data set into two or three partitions linked to intensity (low, medium, and high). Finally, a multiple correspondence analysis (MCA) was applied using the latent variables; it is the counterpart of principal component analysis but for categorical data.

Similar approaches have been developed to identify optimal enzymes and proteins to generate food protein-derived bioactive peptides [49] or for the discovery of specific peptides [21] in the field of bioinformatics. These approaches were based on the generation of in silico data, but both aimed to screen a large batch of proteins and enzymes in the work of Tu et al. [49] and peptides in the works of Siow et al. [21], which are the counterparts of the protein extracts used in our study. Recently, Gauglitz et al. [50] reported the development of a statistical workflow to explore and visualize the similarities and dissimilarities of raw and processed food products. To do this, they used untargeted mass spectrometry (MS data) and molecular networking to reveal molecular changes due to processing. Principal coordinates analysis was used for clustering. Moreover, using beta diversity analysis of food types (yogurt, tea, coffee, meat, and tomato) and their processing, they were able to visualize the molecular relationship among all the samples analyzed. The term β-diversity was first introduced in the field of ecology and corresponds to the ratio between regional and local species diversity.

## 5. Conclusions

The objective of this study was to develop a workflow to screen different origins of protein extracts and identify their potentiality as high quality nutritional culinary aids for recipes for the elderly. As expected, the composition of the protein extracts was linked to their origin and the digestive properties highlighted that animal and dairy proteins released peptides more rapidly, at least in the upper part of the digestive tract. However, not only proteins were targeted (i.e., also minerals, vitamin, etc.). Therefore, the search to reduce variables from 48 to 13 latent variables without losing information was explored in the statistical workflow developed. Such an approach permitted identifying protein extracts capable of satisfying the criteria applied. MCA unveiled complementary protein extracts. Their combination could not only offer a culinary aid, combining micro and macro nutrients, but also high levels of easily digested proteins and essential amino acids.

Such an approach can also be applied to other ingredients and is compatible with the incorporation of technical-functional and/or sensory data. Finally, the addition of culinary aids should be validated through a hedonic study.

## Figures and Tables

**Table 1:** Identification of the different samples and their origin.

Origin	Animal (A)	Dairy (D)	Plant (P)
1	Pork meat broth (hydrolyzed) ~	Cheese powder ^	Fava bean •
2	Pork heart ~	80% serum protein concentrate ^	Fava bean (fermented) •
3	Pork broth ~	60% micellar casein concentrate ^	Hemp *
4	Pork collagen ~	Whey protein ^	Pea *
5	Pork liver ~	Whey protein ^	Pea *
6	Pork liver ~	Calcium caseinate *	Soybean *
7	Chicken broth ♦	Casein rennet *	Soybean *
8	Chicken collagen ♦	Milk protein concentrate *	Pumpkinseed *
9	Chicken meat ♦	Whey protein *	Sunflower *

* Solina; ^ Sodiaal; ♦ Diana Food; ~ Cooperl; • Adria.

**Table 2:** Dry matter (DM) in percentage, macronutrient composition in percentage (protein, total sugar, simple and complex carbohydrates) and micronutrient composition (minerals and vitamins) of animal (A), dairy (D), and plant (P) protein extract. Lipid and protein oxidation values. NA means that the data were not available in triplicate. n.r. means not retrieved.

	Sample	DM (%)	Protein (%)	Total Sugar (%)	Simple Carbohydrates and Oligosaccharides (%)	Complex Carbohydrates (%)	Cr (ppm)	Cu (ppm)	Zn (ppm)	Fe (ppm)	Na (ppm)	Mg (ppm)	K (ppm)	Ca (ppm)	Vitamin C (mg/g)	Vitamin A (µg/g)	TBARS (mg MDA/kg Powder)	Carbonyls (nM Hydrazone/mg Protein)
Animal	A1	96.79 ± 0.86	83.46 ± 0.56	0.85 ± 0.01	1.12 ± 0.05	n.r.	0.28 ± 0.02	2.69 ± 0.14	26.58 ± 0.21	34.96 ± 7.55	12,142 ± 103	194 ± 10	1444 ± 32	549 ± 40	1.35 ± 0.07	151.93 ± 48.12	0.50 ± 0.02	17.24 ± 0.59
	A2	94.39 ± 0.44	66.10 ± 0.36	1.11 ± 0.03	0.74 ± 0.03	0.37 ± 0.04	0.12 ± 0.02	0.12 ± 0.02	1.84 ± 0.22	4.14 ± 0.40	5892 ± 132	428 ± 6	6584 ± 134	600 ± 17	0.99 ± 0.06	n.r.	12.40 ± 0.52	7.11 ± 0.13
	A3	95.98 ± 0.25	85.38 ± 2.36	0.53 ± 0.03	1.05 ± 0.02	n.r.	0.13 ± 0.01	2.26 ± 0.03	34.02 ± 1.13	29.74 ± 0.19	1254 ± 16	203 ± 1	1065 ± 24	1544 ± 40	1.28 ± 0.09	n.r.	1.26 ± 0.13	14.99 ± 0.49
	A4	97.10 ± 0.37	88.60 ± 1.06	0.51 ± 0.01	0.84 ± 0.02	n.r.	1.99 ± 0.34	0.74 ± 0.05	3.56 ± 0.03	17.60 ± 1.25	18,848 ± 3	592 ± 15	6796 ± 81	569 ± 19	1.25 ± 0.23	n.r.	0.33 ± 0.02	16.69 ± 0.41
	A5	91.46 ± 0.50	66.50 ± 1.44	0.95 ± 0.03	1.17 ± 0.15	n.r.	0.49 ± 0.22	27.83 ± 1.67	202.21 ± 3.07	619.75 ± 8.81	8523 ± 373	541 ± 20	5955 ± 408	251 ± 13	0.17 ± 0.12	374.76 ± 2.56	22.21 ± 0.92	23.32 ± 1.64
	A6	92.91 ± 0.17	65.85 ± 0.69	1.70 ± 0.05	0.97 ± 0.10	0.74 ± 0.03	0.01 ± 0.01	0.18 ± 0.03	0.06 ± 0.03	0.13 ± 0.04	6 ± 8	4 ± 2	9 ± 4	3 ± 0	0.58 ± 0.03	381.00 ± 59.98	3.36 ± 0.07	8.57 ± 0.10
	A7	96.31 ± 0.10	61.13 ± 1.76	0.42 ± 0.03	1.56 ± 0.07	n.r.	0.40 ± 0.19	0.23 ± 0.07	39.19 ± 0.70	103.33 ± 44.88	2311 ± 100	535 ± 9	3193 ± 176	6521 ± 282	0.84 ± 0.07	88.89 ± 11.50	2.23 ± 0.13	15.57 ± 0.02
	A8	95.83 ± 0.54	88.19 ± 0.44	0.86 ± 0.10	0.31 ± 0.09	0.54 ± 0.19	1.80 ± 0.06	6.72 ± 0.32	40.44 ± 1.11	201.35 ± 2.94	13,140 ± 156	762 ± 60	39,808 ± 46,613	331 ± 17	0.77 ± 0.15	n.r.	1.24 ± 0.07	10.63 ± 0.19
	A9	94.16 ± 0.25	88.54 ± 1.00	0.48 ± 0.01	0.40 ± 0.04	0.09 ± 0.04	0.24 ± 0.03	0.01 ± 0.01	6.18 ± 0.89	9.20 ± 1.20	9539 ± 283	963 ± 23	8542 ± 1172	306 ± 2	0.67 ± 0.17	n.r.	0.36 ± 0.10	6.76 ± 0.36
Dairy	D1	97.82 ± 0.49	28.19 ± 0.63	9.28 ± 1.91	1.91 ± 0.16	7.37 ± 0.24	0.27 ± 0.01	0.50 ± 0.02	23.27 ± 0.09	9.68 ± 0.34	5850 ± 115	293 ± 1	3510 ± 14	7583 ± 5	0.36 ± 0.09	n.r.	0.22 ± 0.03	1.87 ± 0.17
	D2	92.79 ± 0.66	64.50 ± 0.63	1.61 ± 0.04	1.11 ± 0.18	0.50 ± 0.14	0.41 ± 0.01	2.14 ± 0.10	1.67 ± 0.04	6.89 ± 0.19	21,713 ± 556	68 ± 1	248 ± 9	698 ± 5	0.71 ± 0.10	n.r.	n.r.	8.38 ± 0.97
	D3	95.62 ± 1.83	49.96 ± 2.02	20.82 ± 0.65	2.23 ± 0.13	18.60 ± 0.54	0.63 ± 0.01	0.74 ± 0.03	50.78 ± 0.46	5.51 ± 0.10	1485 ± 28	653 ± 9	6233 ± 68	16,313 ± NA	0.20 ± 0.11	n.r.	0.01 ± 0.02	2.82 ± 0.19
	D4	95.55 ± 0.11	76.19 ± 0.82	1.89 ± 0.32	0.29 ± 0.04	1.61 ± 0.34	0.08 ± 0.01	2.72 ± 0.16	2.32 ± 0.24	19.04 ± 1.08	2426 ± 60	518 ± 11	4825 ± 318	3197 ± 97	0.66 ± 0.08	n.r.	0.48 ± 0.06	9.21 ± 1.12
Dairy	D5	95.57 ± 0.63	85.42 ± 2.15	0.54 ± 0.05	0.32 ± 0.09	0.23 ± 0.14	0.11 ± 0.02	0.25 ± 0.04	0.39 ± 0.04	10.71 ± 2.43	542 ± 22	396 ± 10	527 ± 15	1564 ± 17	1.14 ± 0.08	n.r.	0.30 ± 0.10	11.47 ± 0.38
	D6	95.59 ± 0.49	91.13 ± 0.31	0.18 ± 0.02	0.30 ± 0.10	n.r.	0.06 ± 0.02	0.13 ± 0.03	20.82 ± 1.10	12.76 ± 3.18	49± 6	61 ± 11	44 ± 5	7587 ± 459	0.46 ± 0.04	n.r.	n.r.	4.53 ± 0.62
	D7	88.81 ± 0.28	81.69 ± 0.57	0.06 ± 0.01	0.04 ± 0.01	0.03 ± 0.03	0.14 ± 0.03	0.15 ± 0.03	76.52 ± 0.35	5.37 ± 1.22	52 ± 3	648 ± 9	122 ± 1	17,782 ± 666	1.05 ± 0.07	n.r.	n.r.	2.65 ± 0.16
	D8	93.77 ± 0.44	81.63 ± 0.53	1.21 ± 0.15	1.47 ± 0.34	n.r.	0.27 ± 0.01	0.15 ± 0.01	66.29 ± 1.33	14.57 ± 1.39	6368 ± 99	484 ± 15	1336 ± 66	11,730 ± 66	0.48 ± 0.08	n.r.	n.r.	5.37 ± 0.71
	D9	95.34 ± 1.55	77.75 ± 0.51	2.30 ± 0.21	1.01 ± 0.20	1.29 ± 0.04	0.12 ± 0.02	12.11 ± 1.19	77.71 ± 9.00	104.30 ± 7.98	3511 ± 332	5026 ± 51	4740 ± 1449	3471 ± 157	1.28 ± 0.02	n.r.	0.20 ± 0.10	4.71 ± 0.39
Plant	P1	39.05 ± 0.10	26.15 ± 0.19	9.46 ± 0.29	1.08 ± 0.08	8.39 ± 0.21	0.11 ± 0.01	8.13 ± 0.15	25.39 ± 0.05	26.38 ± 0.23	102 ± 42	661 ± 20	4362 ± 55	236 ± 4	1.02 ± 0.07	3.45 ± 0.07	1.47 ± 0.29	11.63 ± 0.76
	P2	43.07 ± 2.10	24.69 ± 1.88	7.05 ± 1.05	2.78 ± 0.10	4.28 ± 1.10	0.02 ± 0.01	6.29 ± 0.85	23.06 ± 1.46	22.64 ± 2.77	6 ± 2	579 ± 54	4448 ± 459	190 ± 8	1.48 ± 0.14	4.73 ± 0.10	0.57 ± 0.09	5.67 ± 1.03
	P3	92.68 ± 0.64	49.13 ± NA	12.31 ± 0.46	2.58 ± 0.14	9.74 ± 0.59	0.53 ± 0.05	16.45 ± 0.86	92.09 ± 4.16	173.13 ± 14.47	2 ± 0	5733 ± 198	5894 ± 46	1463 ± 81	0.69 ± 0.04	3.04 ± 0.39	7.33 ± 0.33	5.71 ± 0.13
	P4	94.21 ± 0.36	84.79 ± 0.32	2.70 ± 0.06	1.32 ± 0.07	1.38 ± 0.13	0.17 ± 0.01	7.81 ± 0.01	49.29 ± 0.72	125.85 ± 5.31	6322 ± 0	471 ± 8	1744 ± 51	706 ± 23	0.85 ± 0.14	2.10 ± 0.17	2.14 ± 0.18	7.22 ± 0.30
	P5	94.01 ± 0.87	69.89 ± 0.32	1.81 ± 0.02	1.39 ± 0.09	0.42 ± 0.07	0.38 ± 0.01	10.44 ± 0.11	61.54 ± 2.40	140.11 ± 1.78	4815 ± 128	405± 9	2183 ± 32	563 ± 6	1.71 ± 0.21	0.41 ± 0.01	1.14 ± 0.24	7.45 ± 0.76
	P6	92.89 ± 1.18	58.61 ± 0.28	11.65 ± 0.57	0.52 ± 0.01	11.14 ± 0.56	0.61 ± 0.02	5.49 ± 0.14	24.12 ± 0.81	89.71 ± 0.20	68 ± 17	1665 ± 32	5423 ± 128	1598 ± 36	1.03 ± 0.03	0.17 ± 0.09	0.24 ± 0.04	4.96 ± 0.72
	P7	96.24 ± 0.66	76.35 ± 0.97	1.71 ± 0.04	0.67 ± 0.24	1.05 ± 0.27	0.34 ± 0.02	10.10 ± 0.19	23.00 ± 0.90	97.34 ± 2.45	3150 ± 36	180 ± 8	4905 ± NA	405 ± 6	1.24 ± 0.15	0.50 ± 0.04	1.65 ± 0.08	7.45 ± 1.13
	P8	95.63 ± 0.67	57.81 ± 0.53	9.07 ± 0.12	1.02 ± 0.28	8.05 ± 0.26	0.15 ± 0.01	13.54 ± 0.83	87.02 ± 4.16	114.47 ± 6.40	3924 ± 252	5377 ± 446	5945 ± 256	690 ± 42	0.85 ± 0.14	6.19 ± 1.11	3.24 ± 0.06	10.51 ± 0.13
	P9	91.07 ± 0.37	54.77 ± 0.10	12.80 ± 0.68	1.47 ± 0.22	11.33 ± 0.86	0.24 ± 0.04	28.86 ± 2.42	90.54 ± 7.07	85.49 ± 1.39	33 ± 45	4680 ± 310	5753 ± 812	1494 ± 201	1.37 ± 0.06	0.66 ± 0.38	0.36 ± 0.05	3.79 ± 0.05

**Table 3:** Aminogram of protein extract, expressed in g/100 g of dry matter (%DM).

	Sample	Indispensable Amino Acids—IAA (%DM)									Dispensable Amino Acids (%DM)									IAA/Total AA (%)	Total AA (%DM)
		His	Ile	Leu	Lys	Met	Phe	Thr	Trp	Val	Arg	Ala	Asp	Cys	Glu	Gly	Pro	Ser	Tyr		
Animal	A1	1.85	3.30	6.24	6.02	0.70	3.32	3.76	0.96	4.21	5.23	6.09	7.28	0.52	11.89	11.52	6.02	3.27	2.01	36.1	84.18
	A2	2.31	3.38	7.32	5.43	0.66	3.26	3.73	0.82	4.24	2.56	5.50	5.78	0.47	12.65	4.37	4.19	1.81	1.35	44.6	69.83
	A3	1.99	3.23	6.48	6.08	0.31	3.50	3.80	0.87	4.60	5.12	6.52	7.29	0.40	11.06	11.02	7.42	3.23	2.05	36.3	84.98
	A4	2.22	1.54	3.54	4.20	0.13	2.15	2.14	0.11	2.73	6.14	8.31	6.07	0.16	11.48	20.90	11.52	3.24	0.86	21.5	87.46
	A5	1.63	3.77	7.19	5.88	0.62	3.85	3.62	1.81	4.73	0.89	4.52	6.57	0.57	9.86	4.72	4.31	3.02	0.99	48.3	68.55
	A6	1.68	4.09	7.98	2.28	0.84	4.02	2.88	1.26	5.25	1.55	5.02	7.09	0.52	9.89	4.82	3.88	2.25	1.45	45.4	66.74
	A7	1.83	3.12	5.77	5.52	0.20	2.90	3.37	0.84	3.31	3.65	4.51	6.15	0.15	9.90	6.28	3.99	2.65	2.11	40.5	66.26
	A8	1.83	2.73	5.54	5.25	0.49	2.87	3.93	0.35	3.42	5.96	7.12	6.77	0.36	12.14	14.41	7.85	3.06	1.95	30.7	86.02
	A9	3.20	3.68	6.77	6.89	0.61	3.64	4.27	0.56	3.88	4.08	7.22	7.96	0.97	14.25	11.03	5.25	3.19	2.49	37.2	89.95
Dairy	D1	0.95	1.60	2.90	2.21	0.42	1.58	1.24	0.62	1.80	0.92	0.97	2.06	0.85	6.20	0.56	3.42	1.47	1.50	42.6	31.26
	D2	1.12	6.18	5.72	5.99	0.22	1.55	7.25	0.66	5.68	2.72	5.72	9.89	0.51	20.36	1.08	10.98	5.52	1.09	37.3	92.24
	D3	1.26	1.52	4.51	5.87	0.66	2.36	2.31	0.95	1.89	2.79	1.52	4.11	0.92	11.23	1.77	11.89	2.22	1.72	35.8	59.50
	D4	1.27	4.89	9.04	8.82	0.61	2.33	5.62	1.51	4.62	1.68	4.30	5.13	0.97	14.08	1.45	4.23	3.35	1.75	51.2	75.65
	D5	1.65	5.59	9.85	7.73	1.24	2.96	6.34	0.24	5.03	1.86	4.51	8.83	0.47	15.16	1.59	5.20	3.89	2.00	48.3	84.13
	D6	2.76	4.40	9.05	7.15	1.34	4.85	3.86	0.20	5.63	2.51	2.74	6.30	0.37	20.10	1.61	8.99	4.95	3.85	43.3	90.65
	D7	2.53	3.59	8.67	6.69	1.58	4.55	3.30	0.59	4.87	2.32	2.28	6.06	0.33	19.38	1.57	8.99	4.93	4.25	42.0	86.47
	D8	2.40	4.26	8.53	6.67	1.61	4.08	3.91	1.17	5.19	1.51	2.71	6.24	0.67	17.94	1.46	7.96	4.38	3.36	45.0	84.05
	D9	1.61	5.35	9.26	7.74	0.72	2.76	6.44	1.68	4.87	1.52	4.26	9.19	0.98	14.94	1.49	4.69	3.86	2.20	48.4	83.56
Plant	P1	0.98	1.38	2.37	1.89	0.48	1.38	1.09	0.11	1.35	2.46	1.23	2.46	0.62	4.66	1.30	1.59	1.62	1.02	39.4	27.98
	P2	0.89	1.55	3.05	2.05	0.38	1.51	1.11	0.12	1.65	1.15	1.28	2.66	0.52	4.89	1.25	1.59	1.56	1.18	43.3	28.38
	P3	1.40	1.85	3.55	2.09	0.64	2.49	2.24	0.61	5.59	4.50	2.49	5.65	0.59	9.95	2.49	2.52	2.68	1.86	38.4	53.19
	P4	1.98	5.71	9.83	7.86	0.24	2.89	6.14	0.77	5.32	1.98	4.52	8.89	0.55	14.89	1.52	5.18	3.68	1.89	48.6	83.84
	P5	1.98	2.72	7.02	8.89	0.51	4.23	3.42	0.88	3.25	6.42	3.55	10.25	0.45	16.72	3.42	8.12	4.52	2.55	37.0	88.90
	P6	1.78	2.19	5.42	7.42	0.61	3.52	3.12	0.72	2.31	4.89	3.15	9.15	0.42	15.62	2.78	8.13	3.55	2.25	35.2	77.03
	P7	2.42	3.56	8.12	6.85	0.52	5.52	4.52	0.75	3.99	7.05	4.36	9.22	0.48	20.12	4.22	9.12	5.21	2.63	36.7	98.66
	P8	1.37	2.32	4.76	2.30	0.49	3.52	2.36	0.91	2.89	6.08	2.98	6.01	0.42	12.50	3.61	2.50	3.19	2.14	34.7	60.35
	P9	1.64	2.45	4.05	2.06	0.58	3.02	2.65	0.87	3.00	6.52	2.45	5.65	0.59	13.25	3.42	3.06	2.51	1.68	34.2	59.45

**Table 4:** Bioaccessibility and bioactivity of peptides from the digestates after 1 h in the gastric phase (means of three repetitions ± SD). The level of protein is measured by the Biuret method, the peptides by Direct Detect^®^, free NH_2_ by fluorescamine method. Antioxidant bioactivity was evaluated with several methods: ABTS, FRAP, DPPH, expressed as % of inhibition and ORAC, expressed as µmol of Trolox equivalent/mg of peptide digestate.

	Sample	Protein (mg/g Powder)	Peptides (mg/g Powder)	Free NH_2_ (mM Glycine Equivalent/g Powder)	Inhibition of ABTS Radical (%)	Antioxidant Power to Reduce Ferric Ions (% Inhibition)	Inhibition of DPPH Radical (%)	ORAC (µmol Trolox Equivalent/mg Peptide Digestate)
Animal	A1	546 ± 23	541 ± 54	4317 ± 620	26.358 ± 2.09	5.31 ± 0.41	51.57 ± 2.56	0.11 ± 0.07
	A2	478 ± 141	435 ± 26	1858 ± 203	37.99 ± 1.47	6.09 ± 0.66	47.59 ± 1.46	0.16 ± 0.04
	A3	558 ± 83	215 ± 40	191 ± 16	25.23 ± 2.39	5.63 ± 0.47	16.72 ± 11.10	1.13 ± 0.33
	A4	516 ± 26	568 ± 21	1794 ± 259	30.98 ± 0.55	6.27 ± 0.11	60.97 ± 0.56	0.34 ± 0.09
	A5	364 ± 20	407 ± 37	2285 ± 914	77.63 ± 3.73	15 ± 8.69	72.41 ± 3.53	0.09 ± 0.02
	A6	269 ± 30	459 ± 35	2152 ± 370	94.66 ± 2.18	39.83 ± 1.51	79.45 ± 1.73	0.06 ± 0.02
	A7	1064 ± 233	276 ± 23	334 ± 49	24.79 ± 1.00	5.97 ± 0.24	9.15 ± 2.39	0.90 ± 0.28
	A8	536 ± 33	663 ± 180	1942 ± 856	60.39 ± 1.97	16.66 ± 0.25	44.6 ± 3.11	0.06 ± 0.01
	A9	370 ± 25	571 ± 41	3458 ± 604	73.82 ± 5.01	16.41 ± 0.71	62.08 ± 4.61	0.06 ± 0.02
Dairy	D1	800 ± 237	153 ± 7	208 ± 138	15.57 ± 0.61	4.24 ± 0.65	54.22 ± 3.61	0.29 ± 0.19
	D2	722 ± 17	231 ± 77	938 ± 84	15.8 ± 0.52	5.35 ± 0.18	21.72 ± 7.10	0.3 ± 0.07
	D3	483 ± 11	211 ± 19	231 ± 10	18.36 ± 0.30	4.49 ± 0.16	57.87 ± 1.32	0.14 ± 0.03
	D4	901 ± 52	246 ± 17	569 ± 64	15.1 ± 0.70	6.38 ± 0.45	52.89 ± 1.94	0.31 ± 0.23
	D5	1018 ± 38	262 ± 22	393 ± 63	11.45 ± 0.36	0.99 ± 0.22	48.16 ± 2.82	0.6 ± 0.32
	D6	839 ± 99	298 ± 24	318 ± 49	12.84 ± 0.54	2.11 ± 0.21	63.68 ± 9.12	0.27 ± 0.09
	D7	462 ± 74	324 ± 32	506 ± 131	10.78 ± 0.44	1.69 ± 0.144	56.53 ± 2.92	0.24 ± 0.05
	D8	698 ± 32	278 ± 34	367 ± 141	12.68 ± 0.76	1.95 ± 0.72	63.67 ± 3.75	1.02 ± 0.4
	D9	869 ± 51	390 ± 43	967 ± 310	14.13 ± 0.44	3.60 ± 0.22	49.25 ± 3.66	0.38 ± 0.11
Plant	P1	219 ± 13	94 ± 6	438 ± 53	88.71 ± 0.33	42.90 ± 1.02	75.49 ± 1.92	0.02 ± 0.04
	P2	203 ± 18	382 ± 77	408 ± 17	88.84 ± 1.65	43.06 ± 4.33	71.11 ± 3.85	0.11 ± 0.01
	P3	431 ± 12	246 ± 20	534 ± 102	47.63 ± 8.54	11.14 ± 2.91	22.01 ± 14.23	0.29 ± 0.10
	P4	859 ± 33	334 ± 33	464 ± 31	20.95 ± 0.98	3.86 ± 0.22	44.86 ± 1.07	0.29 ± 0.07
	P5	874 ± 59	187 ± 25	406 ± 15	14.61 ± 0.59	2.36 ± 0.03	41.79 ± 0.82	0.32 ± 0.07
	P6	675 ± 92	140 ± 36	367 ± 62	21.86 ± 1.68	9.73 ± 0.35	9.61 ± 9.94	1.60 ± 0.78
	P7	628 ± 183	170 ± 38	645 ± 62	21.69 ± 0.44	9.33 ± 0.48	31.04 ± 3.71	0.17 ± 0.21
	P8	295 ± 29	261 ± 29	289 ± 42	39.63 ± 5.04	29.04 ± 5.78	32.18 ± 8.97	0.26 ± 0.09
	P9	505 ± 26	235 ± 16	608 ± 62	82.09 ± 3.67	15.51 ± 3.86	38.15 ± 4.87	0.08 ± 0.04

**Table 5:** Bioaccessibility and bioactivity of peptides from the intestinal digestates after 3 h of digestion (means of 3 repetitions ± SD). The level of protein is measured by the Biuret method, the peptides by Direct Detect^®^, free NH_2_ by fluorescamine method. Antioxidant bioactivity was evaluated with several methods: ABTS, FRAP, DPPH, expressed as % of inhibition and ORAC, expressed as µmol of Trolox equivalent/mg of peptide digestate.

	Sample	Protein (mg/g Powder)	Peptides (mg/g Powder)	Free NH_2_ (mM Glycine Equivalent/g Powder)	Inhibition of ABTS Radical (%)	Antioxidant Power to Reduce Ferric Ions (% Inhibition)	Inhibition of DPPH Radical (%)	ORAC (µmol Trolox Equivalent/mg Peptide Digestate)
Animal	A1	496 ± 80	839 ± 151	6199 ± 1039	15.71 ± 1.17	3.99 ± 0.35	44.64 ± 1.64	0.15 ± 0.07
	A2	250 ± 22	670 ± 29	3418 ± 640	26.62 ± 0.92	5.11 ± 0.21	48.87 ± 0.49	0.46 ± 0.11
	A3	551 ± 47	733 ± 63	2454 ± 398	30.16 ± 1.07	7.00 ± 0.29	51.30 ± 0.63	0.03 ± 0.01
	A4	461 ± 32	859 ± 59	2642 ± 332	18.28 ± 1.23	4.66 ± 0.27	53.44 ± 2.83	1.26 ± 0.33
	A5	271 ± 19	660 ± 54	3257 ± 382	51.42 ± 3.59	17.45 ± 1.07	65.20 ± 2.14	0.05 ± 0.04
	A6	187 ± 35	739 ± 76	3791 ± 773	70.33 ± 3.82	26.11 ± 1.29	66.89 ± 2.02	0.10 ± 0.01
	A7	549 ± 59	546 ± 48	1661 ± 397	21.02 ± 1.94	6.73 ± 0.37	56.00 ± 1.27	0.40 ± 0.11
	A8	418 ± 31	1213 ± 324	2362 ± 265	35.20 ± 1.37	10.55 ± 0.36	33.00 ± 2.21	0.10 ± 0.06
	A9	341 ± 87	870 ± 181	5142 ± 392	43.77 ± 1.08	10.47 ± 0.65	59.93 ± 1.71	0.17 ± 0.08
Dairy	D1	763 ± 91	333 ± 27	798 ± 366	5.76 ± 6.41	5.13 ± 0.83	25.65 ± 1.86	0.02 ± 0.02
	D2	568 ± 85	464 ± 27	2559 ± 844	16.17 ± 0.55	5.91 ± 0.44	33.16 ± 5.98	0.12 ± 0.04
	D3	335 ± 8	530 ± 33	2188 ± 89	14.37 ± 0.29	4.59 ± 0.07	20.82 ± 1.54	0.06 ± 0.04
	D4	525 ± 53	604 ± 62	2486 ± 1415	18.33 ± 0.65	13.80 ± 0.46	44.11 ± 1.01	0.11 ± 0.07
	D5	563 ± 91	837 ± 74	3529 ± 651	14.38 ± 0.86	2.14 ± 0.11	34.96 ± 3.52	0.13 ± 0.05
	D6	505 ± 29	854 ± 50	3433 ± 678	11.26 ± 0.53	2.25 ± 0.19	35.49 ± 4.46	0.07 ± 0.01
	D7	412 ± 159	861 ± 63	5146 ± 430	10.89 ± 0.35	2.03 ± 0.09	24.96 ± 0.78	0.04 ± 0.04
	D8	452 ± 24	786 ± 40	3004 ± 456	13.21 ± 0.31	2.87 ± 0.20	31.98 ± 1.45	0.19 ± 0.08
	D9	507 ± 37	819 ± 106	4205 ± 655	16.58 ± 1.60	4.45 ± 0.16	44.03 ± 1.62	0.17 ± 0.02
Plant	P1	144 ± 21	253 ± 17	1000 ± 192	80.10 ± 0.66	38.49 ± 0.78	76.62 ± 1.87	0.09 ± 0.01
	P2	186 ± 189	771 ± 48	650 ± 91	67.21 ± 5.70	43.21 ± 1.93	71.85 ± 0.90	0.17 ± 0.04
	P3	325 ± 22	586 ± 44	2511 ± 279	74.90 ± 6.27	22.60 ± 1.49	38.91 ± 7.70	0.13 ± 0.03
	P4	541 ± 67	719 ± 95	2158 ± 94	25.30 ± 1.24	6.46 ± 0.16	45.33 ± 0.91	0.22 ± 0.04
	P5	502 ± 36	554 ± 56	3264 ± 106	22.16 ± 0.36	6.04 ± 0.13	39.38 ± 1.62	0.14 ± 0.02
	P6	418 ± 15	423 ± 40	3059 ± 156	28.80 ± 1.11	13.33 ± 0.44	21.81 ± 5.78	0.51 ± 0.52
	P7	463 ± 109	506 ± 104	4968 ± 242	35.26 ± 0.96	13.85 ± 0.90	47.83 ± 2.61	0.36 ± 0.06
	P8	310 ± 20	808 ± 194	2372 ± 354	39.08 ± 6.24	22.60 ± 1.49	39.84 ± 3.22	0.08 ± 0.12
	P9	300 ± 18	628 ± 48	2403 ± 325	82.56 ± 4.56	82.78 ± 2.08	66.40 ± 4.14	0.06 ± 0.03

**Table 6:** Summary table of the results obtained after the creation of latent variables by HCA and then class variables by the k-means method. Fifteen latent variables created from the 48 initial variables, with two or three levels (high, medium, or low).

HCA		k-Means		
Variable	Latent Variable	High (H)	Low (L)	Medium (M)
Direct detect	Peptides_G	A1; A3; A5; A6; A7; A8; A9	A2; A4; D1; D2; D3; D4; D5; D6; D7; D8; D9; P1; P2; P3; P4; P5; P6; P7; P8; P9	
Fluorescamine				
%ABTS	Antiox_G	A5; A7; A8; A9; P2; P3; P4; P9	A1; A2; A3; A4; A6; D1; D2; D3; D4; D5; D6; D7; D8; D9; P1; P5; P6; P7; P8	
%FRAP				
%DPPH				
Biuret	BiuretORAC_G	A2; A4; D1; D2; D3; D5; D8; D9; P1; P7; P8	A1; A3; A5; A6; D4; D6; D7; P5; P6; P9	A7; A8; A9; P2; P3; P4
ORAC				
Direct detect	Peptides_I	A1; A4; A6; A8; A9; D5; D6; D7; D8; D9	A7; D1; P1; P2	A2; A3; A5; D2; D3; D4; P3; P4; P5; P6; P7; P8; P9
Fluorescamine				
%ABTS	Antiox_I	A6; P1; P2; P9	A8; D1; D2; D3; D5; D6; D7; D8; P5; P6	A1; A2; A3; A4; A5; A7; A9; D4; D9; P3; P4; P7; P8
%FRAP				
%DPPH				
Biuret	BiuretORAC_I	A1; A3; A4; A7; D1; D2; D4; D5; D6; D8; D9; P4; P5; P6; P7	A2; A5; A6; A8; A9; D3; D7; P1; P2; P3; P8; P9	
ORAC				
%Protein	Prot_HFDSEP	A1; A3; A4; A8; A9; D2; D5; D6; D7; D8; D9; P4; P5; P6; P7	A2; A5; A6; A7; D1; D3; D4; P1; P2; P3; P8; P9	
Histidine				
Phenyalanine				
Aspartic acid				
Serine				
Glutamic acid				
Proline				
Threonine	TIVLK	D2; D5; D4; D9; P4	A1; A2; A3; A5; A6; A7; A8; A9; D6; D7; D8; P5; P6; P7	A4; D1; D3; P1; P2; P3; P8; P9
Isoleucine				
Valine				
Leucine				
Lysine				
Ca	Ca_YM	D3; D6; D7; D8	A1; A2; A3; A4; A5; A6; A7; A8; A9; D1; D2; D4; D5; D9; P1; P2; P3; P4; P5; P6; P7; P8; P9	
Tyrosine				
Methionine				
Total sugar	Carbohydrates_Mg	D1; D3; P1; P2; P3; P6; P8; P9	A1; A2; A3; A4; A5; A6; A7; A8; A9; D2; D4; D5; D6; D7; D8; D9; P4; P5; P7	
Complex carbohydrates				
Simple carbohydrates and oligosaccharides				
Mg				
Cu	Micro-nutrients	A5; D1; D9; P3; P5; P8; P9	A1; A2; A3; A4; A6; A7; A8; A9; D2; D3; D4; D5; D6; D7; D8; P1; P2; P4; P6; P7	
Zn				
Fe				
Vitamin A	VitA_Ox	A5; A6	A1; A2; A3; A4; A7; A8; A9; D1; D2; D3; D4; D5; D6; D7; D8; D9; P1; P2; P3; P4; P5; P6; P7; P8; P9	
TBARS				
Carbonyles				
Cysteine	C and W	A9; D1; D3; D4; D9	A1; A2; A3; A5; A6; A7; D2; D8; P3; P4; P5; P6; P7; P8; P9	A4; A8; D5; D6; D7; P1; P2
Tryptophan				
Cr	CrKNa_GA	A4; A8	A1; A2; A3; A5; A6; A7; A9; D1; D2; D3; D4; D5; D6; D7; D8; D9; P1; P2; P3; P4; P5; P6; P7; P8; P9	
K				
Na				
Glycine				
Alanine				
Vitamin C	VitC_R	A1; A2; A3; A4; A7; A8; D5; D7; D9; P1; P2; P3; P5; P6; P7; P8; P9	A5; A6; A9; D1; D2; D3; D4; D6; D8; P4	
Arginine				
